# Prevalence of depression, anxiety and stress during the COVID-19 pandemic: a cross-sectional study among Palestinian students (10–18 years)

**DOI:** 10.1186/s40359-021-00688-2

**Published:** 2021-11-30

**Authors:** Eqbal Radwan, Afnan Radwan, Walaa Radwan, Digvijay Pandey

**Affiliations:** 1 Department of Biology, Faculty of Science, Islamic University of Gaza, Gaza Strip, Palestine; 2 Directorate of Education-East Gaza, Ministry of Education and Higher Education, Gaza Strip, Palestine; 3 Faculty of Education, Islamic University of Gaza, Gaza Strip, Palestine; 4 Faculty of Education, Ummah Open University, Gaza Strip, Palestine; 5 Department of Technical Education, Dr. A.P.J. Abdul, Kalam Technical University Uttar Pradesh, Lucknow, India

**Keywords:** COVID-19, Stress, Depression, Anxiety, DASS-21 scale, Gaza Strip, Palestine

## Abstract

**Background:**

The COVID-19 pandemic considers a threat to students’ well-being and mental health. The current descriptive cross-sectional study aims to identify psychological distress among school students during the lockdown period.

**Methods:**

This study was carried out in a sample of 420 primary and secondary school students from June 10 to July 13, 2020, in the Gaza Strip in Palestine. Data was collected using an online questionnaire that included informed consent, socio-demographic questions, and a psychometric scale (DASS-21).

**Results:**

The results revealed that most students experienced moderate to severe levels of anxiety (89.1%) and depression (72.1%), whereas less than half of them (35.7%) experienced moderate to severe stress. Stress, anxiety and depression scores were significantly different across gender, age groups, family size, and family's economic status. The results showed that gender (β = -0.174, *p* < 0.001), age (β = −0.155, *p* = 0.001) and economic level of family (β = −0.147, *p* = 0.002) were negative predictors correlated with stress. Family size (β = 0.156, *p* = 0.001) played a positive role in stress. It was found that gender (β = −0.105, *p* = 0.031), age (β = −0.135, *p* = 0.006) and economic level of family (β = −0.136, *p* = 0.005) were negative predictors correlated with anxiety, whereas family size (β = 0.139, *p* = 0.004) played a positive role in anxiety. For depression, gender (β = −0.162, *p* = 0.001), age (β = −0.160, *p* = 0.001) and economic level of family (β = −0.131, *p* = 0.007) were negative predictors correlated with depression, whereas family size (β = −0.133, *p* = 0.006) was found to be a positive predictor. Concerns about the influence of COVID-19 on economic, education, and daily life were positively correlated to the levels of depression, anxiety and stress, whereas the availability of social support was negatively correlated.

**Conclusion:**

The development of a health protocol for influenced students is urgently needed to maintain them remain resilient during dangerous times.

## Background

The COVID-19 pandemic has rapidly spread in most countries and brought unexpected health, economic, social, education, psychological consequences [1–3]. Emergencies times can affect the public's well-being, protection, and health (causing confusion, insecurity, stigma, and emotional isolation) and communities (causing school closures, work loss, food insecurity, deficient distribution of necessities, and inadequate resources for medical response).

During a health crisis, people tend to suffer the panic and stress of being infected with the disease resulting in depression, stress and anxiety [4–6]. People facing health diseases with no treatments or vaccines will result in feeling panic and making them stressed, depressed and anxious. Individuals have experienced psychological distress and mental health issues as a result of the outbreak of the COVID-19 pandemic [7–9]. Therefore, the mental health of people should be taken into consideration and timely action to maintain health during the pandemics. The World Health Organization (WHO) has published important guidelines to address psychological problems, which in the culminating may reach to suicide [10, 11]. In the study of Wang et al. [12] and Qiu et al. [13], they mentioned that the Chinese experienced a high level of psychological distress in the time of the COVID-19 era. Most studies carried out in different countries found high psychological difficulties among participants during the COVID-19 crisis [14–18].

During the current pandemic, the psychological status of the school students was strongly affected due to the closing of schools and other educational consequences resulting from this sudden closure. According to [19], more than one-fifth of junior high and high school students' mental health was significantly influenced during the COVID-19 pandemic. In addition, Thakur [20] and Gazmararian et al. [21] showed that the COVID-19 pandemic has had a negative psychological effect on school students, and reported the prevalence of anxiety, depression, and stress among students. Moreover, Shepherd et al. [22] found that the majority of students confirmed that their mental health had deteriorated as a result of the COVID-19 restrictions, expressing anxiety, panic, stress and fear. In another study conducted in Poland, it was reported that students were exposed to a significant number of stressful situations during the COVID-19 pandemic such as disruptions in academic sessions, diminished social and family interactions, particularly for foreign-language students who remained in the place where they stay away from their families and homes [23]. During the first phase of the UK lockdown (April-June 2020), a convenience sample of 168 school-aged children aged 7.6–11.6 years in the UK found a significant increase in depression symptoms compared to the initial assessment 18 months prior to the lockdown [24].

Preventive and control procedures have been adopted to contain the widespread COVID-19. Despite the importance of these procedures, they have long and short term consequences for the well-being and mental health of the individuals [25]; Spoorthy et al. [26]. These negative impacts may translate into unhealthy behaviours (e.g. excessive substance use), emotional reactions (e.g. depression, distress, anxiety, fear, etc.), and non-compliance with the public health procedures (e.g. home quarantine and confinement, and vaccination) in the population [1, 27].

The COVID-19 pandemic was first discovered in Palestine on March 5, 2020, in the West Bank. As of July 13, 2020, there were 73 infected cases in the Gaza Strip, with 63 cases having recovered [28]. In the Gaza Strip, the Ministry of Education and Higher Education (MOEHE) has implemented closing all schools to control the widespread of COVID-19 and avoid the large scale of infection among school students. During the closing of schools, students were instructed to complete their learning through digital platforms [29] and adopt preventive measures such as staying home, wearing gloves and face masks, regularly washing hands, and avoiding public places and gatherings.

The school closures influence students differently with respect to their age, family size, socioeconomic status, residing place, or having a member who works in the medical field etc. For example, a student who has daily necessities may not be as distressed as a student who does not have. In addition, a student who has a member who works in the frontline with COVID-19 (such as a nurse, doctors, etc.) would be more distressed than others who have not. Students around the world are also experiencing distress due to unlimited periods of school closure, the uncertainty of examinations, ability to work, and with respect to the availability of internet services and using digital platforms, etc. For instance, a student who is unable to access lessons online would be more distressed than others who easily access internet services and engage in lectures and lessons [3, 29, 30].

Because school closure was implemented in the Gaza Strip during the COVID-19 period, distress experienced (stress, anxiety and depression) by school students during the COVID-19 pandemic has not been previously investigated in Palestine. Therefore, the present study aimed to investigate psychological distress among school students in the time of lockdown period. This study is the first attempt of its kind to fill the sensitive gap and help policymakers for the management of students' mental health.

## Methods

### Study setting and design

The current descriptive cross-sectional study was carried out from June 10 to July 13, 2020, the three months after the schools’ closure due to the outbreak of the COVID-19 pandemic in the Gaza Strip in Palestine. Due to imposed lockdown, the required data was collected through an online questionnaire, which was distributed on different platforms of social media such as Facebook, WhatsApp, Viber, Virtual Classrooms, or Instagram. An online survey tool (Google Forms) was used to conduct the survey. There were no incentives or benefits offered in exchange for taking part. During the survey, students were asked to offer informed consent and then were asked this question: "Are you willing to participate in this study freely and voluntarily?". If the student replied "no", a blank survey form was instantly sent to them. If the participant responded “yes”, he or she was granted access to the survey form. The questionnaire was translated into the Arabic language for data collection. School children above the age of 16 who understood the content of the questionnaire and agreed to participate in the study were asked to complete it on their own by clicking on the link. The students under the age of 16 years were instructed to have their guardians complete the questionnaire on their behalf after explaining and clarifying the questions. One of the student's parents was one of the guardians who answered the questions on their behalf (mother or father). In the event that one or both of them were absent due to death or other circumstances, his sister, brother, or other relative answered on his behalf.

### Sample technique and procedure

This study used a multistage random sampling method, in which samples are selected in stages using smaller sampling units. Rafah, Khan Younis, Deir Al-Balah, Gaza and North Gaza are the five governorates that make up the Gaza Strip. All Palestinian students are studying in private schools, public schools, or UNRWA schools. Private schools and public schools are supervised by the Palestinian Ministry of Education whereas UNRWA schools are supervised by the United Nations Relief and Works Agency for Palestine Refugees in the Near East (UNRWA). A total of 576,951 students have been registered in all schools. We used a simple random sampling and lottery approach to choose the schools from five governorates.

### Sample size determination and sampling procedures

This study had one objective, namely, to investigate psychological distress among school students in the time of lockdown period due to the outbreak of the COVID-19 pandemic. Because COVID-19 is a newly developing disease with limited data at the national level, the sample size was estimated using a single population proportion calculation by assuming a prevalence of 54%, 95% of the confidence level and 5% of the margin of error. We used the p-value from previous studies [12, 31] which were conducted during the same pandemic (54.0%). The calculated sample size of this study was 382 participants. By adding a tolerable non-response rate (10%), the total sample size was 420 participants.

$$\begin{aligned} {\text{P}} & = 0.{54} ,\;{\text{where}}\;{\text{n}} = {\text{required}}\;{\text{sample}}\;{\text{n}} \\ & = \left( {{\text{Z}}_{{\alpha /{2}}} } \right)^{{2}} [\left( {{\text{p}}*{\text{q}}} \right)/\left( {{\text{d}}^{{2}} } \right),\;{\text{p }} = \, 0.{54} \\ & = \left( {{1}.{96}} \right)\;\left( {{1}.{96}} \right)\;\left( {0.{54}} \right)\;\left( {0.{46}} \right)/\left( {0.0{5}} \right)\;\left( {0.0{5}} \right) = {382} \\ \end{aligned}$$

where, Z is the reliability coefficient of standard error at a 95% confidence interval = 1.96; p = prevalence = 0.54; q = (1 − p) = (1 − 0.54) = 0. 46; d = (margin of error) = 0.05; N = non-response rate 10% = 38; The final total sample = 382 + 38 = 420.

A total of 420 students from primary and secondary schools responded to the online questionnaire, which was distributed via social media platforms during the school closure period.

### Eligibility criteria of selection of participants

1. Recording and studying in a private, public, or UNRWA school during conducting this study.
2. Aged between 10 to 18 years.
3. Live in one of the governorates of the Gaza Strip.

### Measures

Data was collected using a structured and self-related survey questionnaire that included informed consent, socio-demographic questions, and a psychometric scale (DASS-21) to assess DAS. The COVID-19 situation was specifically mentioned in the informed consent, as was our interest in assessing psychological status during the COVID-19 pandemic.

#### Socio-demographic measures

Socio-demographic data were collected during the survey including gender, age, economic level of family and size of the family. The economic level of the family was classified into three categories (low, moderate, and high level) and was based on the monthly family income according to the indicator of the Palestinian Central Bureau of Statistics (PCBS) for households in the Gaza Strip. Family size was also classified into three categories (small, medium, and large). The small family consists of less than 5 individuals; Medium: 5–7 individuals; and Large: more than 7 individuals. In addition, they were also inquired about COVID-19-related stressors such as concern about the economic and educational influences of COVID-19, impact COVID-19 on their daily life and the availability of social support.

#### Depression, anxiety and stress scale (DASS-21)

The DASS-21 is widely used to assess psychological distress experienced by participants in the preceding week [32, 33]. The validated Arabic version was used [34] in this study. This self-reported tool consists of 21 items, with 7 items under each of the three subscales (i.e., depression, anxiety, and stress). Items are scored on a 4-point Likert scale ranging from 0 (Did not apply to me at all) to 3 (Applied to me very much, or most of the time). Sum scores are calculated by multiplying the total number of scores for each subscale (i.e., depression, anxiety, and stress) by 2. To categorize levels of DAS, predefined limits for mild, moderate to severe or extremely severe symptoms levels were used interpreted as shown in Table 1 [32]. In the current study, Cronbach's alpha coefficients (95% confidence interval) for the depression, anxiety, and stress subscales were found to be 0.88 (95% CI = 0. 87–0.90), 0.91 (95% CI = 0.90–0.92), and 0.93 (95% CI = 0.92–0.94), respectively, and the overall DASS-21 scale was found to have a high reliability (Cronbach’s alpha = 0.96).

**Table 1:** The severity ratings used to interpret DASS-21

Severity	Depression	Anxiety	Stress
Normal	0–9	0–7	0–14
Mild	10–13	8–9	15–18
Moderate	14–20	10–14	19–25
Severe	21–27	15–19	26–33
Extremely severe	+ 28	+ 20	+ 34

### Statistical analysis

SPSS version 22 was used to analyze the data. Descriptive statistics including frequency, percentage, mean and standard deviation were used to describe the socio-demographic characteristics of the participants, depression, anxiety and stress. One-way analysis of variance (ANOVA) and independent Samples T-test was used to compare mean scores based on demographic variables. Before conducting the Samples T-test, assumptions were carefully checked. The scale of measurement, random sampling, normality of data distribution, adequacy of sample size, and equality of variance are all assumptions that were satisfied when conducting a Samples T-test. With respect to the ANOVA test, the assumptions were also satisfied (normality, independence, and equal variance). Normal distribution of data was examined using the Shapiro–Wilk test as well as Q-Q plots. Levene's test was used to determine the homogeneity of variance. A Chi-square test was applied to test independence. To report the magnitude of differences between groups, eta squared (η^2^) was reported as a measure of effect size. Effect sizes were reported as either small (η^2^ = 0.01), medium (η^2^ = 0.06), or large (η^2^ = 0.14) [35]. Multiple linear regression analyses were conducted to identify the unique contribution of relevant predictors on the DASS-21 stress, anxiety and depression subscale scores. Spearman's correlation coefficient (r) was applied to assess the association between depression, anxiety and stress level and COVID-19-related stressors (stressors related to economic, daily-life, and delays in academic activities). The statistical significance level was set at p < 0.05 (two-sided). All tests of associations were carried out at a level of significance of < 0.05 and 95% confidence Interval.

### Ethical considerations and informed consent to participate

Students confirm that they have given their informed consent to participate in this study. The Ethics Committee of the MOEHE approved the procedures before applying the questionnaire. The procedures used in this study were compliant with the Declaration of Helsinki's guidelines for research involving human subjects. After clarifying the study’s objective, all participants above the age of 16 years were provided with informed consent for participation and publication. The informed consent statement for students under the age of 16 years was obtained from their guardians. The students’ guardians were one of the student's parents (mother or father). In the event that one or both of them were absent due to death or other circumstances, his sister, brother, or other relative answered on his behalf.

## Results

### Socio-demographic Characteristics of students

Four hundred and twenty (N = 420) students completed the online questionnaire. More than half of the students (67.4%) were females and 32.6% were males. The age frequency showed that 75.0% of students were from 10 to 14 years. In addition, about 65.0% confirmed that the economic level of their family is moderate. Two-third of students (66.2%) have a medium family and only 7.4% have a small family (Table 2).

**Table 2:** Students' socio-demographic characteristics (N = 420)

Variable	Category	N	%
Gender	Female	283	67.4
	Male	137	32.6
Age	10–14	315	75.0
	15–18	105	25.0
Family economic level*	Low	114	27.1
	Moderate	273	65.0
	High	33	7.9
Family size**	Small	31	7.4
	Medium	278	66.2
	Large	111	26.4
Total		420	100.0

*Based on PCBS indicator for households in the Gaza Strip

**Small family: consists of less than 5 individuals; Medium: 5–7 individuals; and Large: more than 7 individuals

More than half of the participants (51.9%) had normal scores on the stress subscale, but 12.4% had scores in the mild range, and 13.3% classified as severe. On the anxiety subscale, only 1.6% had scores in the mild range. Severe symptoms of stress were experienced by 13.3%, which is closer to the 11.7% who experienced severe symptoms of anxiety and 9.0% who experienced severe symptoms of depression (Table 3).

**Table 3:** Participants' performance on the three subscales of the Depression, Anxiety and Stress Scale—21 Items (DASS-21)

	Stress^a^	Anxiety^a^	Depression^a^
Score, Mean ± SD	16.94 ± 5.67	20.30 ± 5.53	17.80 ± 5.27
Categories, N (%)			
Normal	218 (51.9)	39 (9.3)	46 (11.0)
Mild	52 (12.4)	7 (1.6)	71 (16.9)
Moderate	39 (9.3)	136 (32.4)	161 (38.3)
Severe	56 (13.3)	49 (11.7)	38 (9.0)
Extremely severe	55 (13.1)	189 (45.0)	104 (24.8)

^a^Subscales of the DASS

Females' mean stress, anxiety, and depression levels were 20.86, 18.20, and 19.34, respectively (Table 4). Males' stress, anxiety, and depression mean values were 18.14, 14.52, and 15.44, respectively. Female students had higher mean values than male students on stress (*p* = 0.006; η^2^ = 0.018), anxiety (*p* = 0.002; η^2^ = 0.023), and depression (*p* = 0.001; η^2^ = 0.026). Males had mild stress and moderate anxiety, while females had moderate stress and severe anxiety. Moderate depression was recorded by both females and males.

**Table 4:** Gender differences

Variable	Gender	N	Mean	SD	Level	t	*p* value	Eta squared
Stress	Female	283	20.86	4.71	Moderate	2.77	0.006	0.018
	Male	137	18.14	4.74	Mild			
Anxiety	Female	283	18.20	5.77	Severe	3.19	0.002	0.023
	Male	137	14.52	5.44	Moderate			
Depression	Female	283	19.34	5.57	Moderate	3.31	0.001	0.026
	Male	137	15.44	5.78	Moderate			

The study showed that the mean stress, anxiety, and depression levels for students aged 15 to 18 years were 20.76, 17.98, and 19.12, respectively (Table 5). The mean values for stress, anxiety, and depression in students aged 10–14 years were 17.60, 14.04, and 14.84, respectively, with 5.0, 5.2, and 5.8 as standard deviations. Students aged 15–18 years had higher mean values than those aged 10–14 years on depression (*p* = 0.001; η^2^ = 0.026), stress (*p* = 0.003; η^2^ = 0.021), and anxiety (*p* = 0.001; η^2^ = 0.022). Moderate depression was identified by both age groups. Moderate stress and severe anxiety were identified by the older students.

**Table 5:** Age differences

Variable	Age	N	Mean	SD	Level	t	*p* value	Eta squared
Stress	10–14	106	17.60	5.0	Mild	2.97	0.003	0.021
	15–18	314	20.76	4.6	Moderate			
Anxiety	10–14	106	14.04	5.2	Moderate	3.25	0.001	0.022
	15–18	314	17.98	5.8	Severe			
Depression	10–14	106	14.84	5.5	Moderate	3.36	0.001	0.026
	15–18	314	19.12	5.8	Moderate			

Table 6 shows how stress, anxiety, and depression vary by level of income family. For students from low, moderate, and high-income families, the mean values for stress were 21.08, 19.96, and 16.18, respectively. Students from low and moderate-income families showed moderate stress, whereas those from high-income families showed normal levels of stress (*p* = 0.033; η^2^ = 0.016). On anxiety, the scores for students from low, moderate, and high-income families were 19.60, 16.38, and 13.14, respectively. Students from low and middle-income families had severe anxiety, while students from high-income families had moderate anxiety (*p* = 0.005; η^2^ = 0.025). In terms of depression, students from low, moderate, and high-income families reported mean values of 20.28, 17.64, and 13.86, respectively. Students from high-income families had normal levels of depression, while students from moderate-income families had moderate levels (*p* = 0.01; η^2^ = 0.022). Furthermore, students from low-income families expressed severe depression. In addition, since the sample sizes were unequal, a post hoc comparison was conducted using Games Howell. The difference in stress (Md = 2.45, *p* = 0.015), anxiety (Md = 3.23, *p* = 0.017) and depression (Md = 3.20, *p* = 0.015) levels between students from low-income families and those from high-income families was low significant. In addition, students from low-income families and those from moderate-income families (Md = 1.61, *p* = 0.04) showed a low significant difference in anxiety.

**Table 6:** Economic level differences

Variable	Level of income family	N	Mean	SD	Level	F	*p* value	Eta squared
Stress	Low	114	21.08	4.2	Moderate	3.44	0.033	0.016
	Moderate	273	19.96	4.9	Moderate			
	High	33	16.18	4.2	Mild			
Anxiety	Low	114	19.60	6.0	Severe	5.33	0.005	0.025
	Moderate	273	16.38	5.4	Severe			
	High	33	13.14	5.6	Moderate			
Depression	Low	114	20.28	5.6	Severe	4.67	0.010	0.022
	Moderate	273	17.64	5.6	Moderate			
	High	33	13.86	5.6	Mild			

Table 7 shows the differences in stress, anxiety, and depression among students based on their family size. For students from small, medium, and large families, the mean values for stress were 16.64, 19.78, and 21.34, respectively. Moderate stress was reported by students from medium and large families, while mild stress was reported by students from small families (*p* = 0.044; η^2^ = 0.015). The mean anxiety scores for students from small, medium, and large families were 13.66, 16.18, and 19.98, respectively. Students from medium and large families had severe anxiety, while students from small families had moderate anxiety (*p* = 0.003; η^2^ = 0.028). Students from small, medium and large families recorded 14.12, 17.25, and 20.52 as their mean depression ratings, respectively. With regards to depression, there was a low significant difference between the groups (*p* = 0.009; η^2^ = 0.023). It was clear that all of the students were depressed to a moderate level. The results revealed a low significant difference between students from small and moderate families in stress (Md = 2.35, *p* = 0.023), anxiety (Md = 3.15, *p* = 0.027) and depression (Md = 3.19, p = 0.023). Also, a low significant difference was observed in anxiety (Md = 1.89, *p* = 0.013) and depression (Md = 1.49, *p* = 0.049) levels between students from small and large families.

**Table 7:** Family size differences

Variable	Family size	N	Mean	SD	Level	F	*p* value	Eta squared
Stress	Small	31	16.64	4.2	Mild	3.14	0.044	0.015
	Medium	278	19.78	4.9	Moderate			
	Large	111	21.34	4.1	Moderate			
Anxiety	Small	31	13.66	5.7	Moderate	5.89	0.003	0.028
	Medium	278	16.18	5.4	Severe			
	Large	111	19.98	6.0	Severe			
Depression	Small	31	14.12	5.6	Moderate	4.81	0.009	0.023
	Medium	278	17.52	5.6	Moderate			
	Large	111	20.52	5.6	Moderate			

### Factors affecting the depression, anxiety and stress of school students during the COVID-19 pandemic

Table 8 showed that gender (β = −0.174, *p* < 0.001), age (β = −0.155, *p* = 0.001) and economic level of family (β = −0.147, *p* = 0.002) were negative predictors correlated with stress. Family size (β = 0.156, *p* = 0.001) played a positive role in stress. Similarly to stress, it was found that gender (β = −0.105, *p* = 0.031), age (β = −0.135, *p* = 0.006) and economic level of family (β = −0.136, *p* = 0.005) were negative predictors correlated with anxiety, whereas family size (β = 0.139, *p* = 0.004) played a positive role in anxiety. For depression, gender (β = −0.162, *p* = 0.001), age (β = −0.160, *p* = 0.001) and economic level of family (β = −0.131, *p* = 0.007) were negative predictors correlated with depression, whereas family size (β = −0.133, *p* = 0.006) was found to be a positive predictor.

**Table 8:** Multiple linear regression model predicting students’ stress, anxiety and depression (n = 420)

	Unstandardized Coefficients		Standardized Coefficients	t	*p*	95% Confidence Interval for B	
	B	SE	β			Lower Bound	Upper Bound
Stress							
Gender	−2.097	.581	−.174	−3.607	< 0.001	−3.240	−.954
Age	−2.025	.631	−.155	−3.208	0.001	−3.267	−.784
Family economic level	−1.490	.489	−.147	−3.045	0.002	−2.453	−.528
Family size	1.612	.498	.156	3.239	0.001	.634	2.590
Anxiety							
Gender	−1.237	.573	−.105	−2.159	0.031	−2.364	−.111
Age	−1.717	.618	−.135	−2.778	0.006	−2.933	−.502
Family economic level	−1.343	.479	−.136	−2.807	0.005	−2.284	−.402
Family size	1.393	.487	.139	2.861	0.004	.436	2.351
Depression							
Gender	−1.820	.542	−.162	−3.356	0.001	−2.885	−.754
Age	−1.946	.587	−.160	−3.314	0.001	−3.100	−.792
Family economic level	−1.235	.457	−.131	−2.706	0.007	−2.133	−.338
Family size	1.272	.465	.133	2.736	0.006	.358	2.185

### Correlation between the COVID-19-related stressors and the depression, anxiety and stress of school students

Table 9 showed that concerns about the COVID-19 pandemic's economic consequences were positively correlated to the levels of depression (r = 0.236, *p* < 0.001), anxiety (r = 0.153, *p* < 0.001) and stress (r = 0.159, *p* < 0.001) in school students. Furthermore, concern about educational influences and was positively correlated with depression (r = 0.310, *p* < 0.001), anxiety (r = 0.150, *p* < 0.001) and stress levels (r = 0.160, *p* < 0.001). The results showed that concern about the COVID-19's impact on daily life was also positively correlated with depression (r = 0.160, *p* < 0.001), anxiety (r = 0.163, *p* < 0.001) and stress levels (r = 0.142, *p* < 0.001). Moreover, the findings revealed a negative correlated between social support and symptoms of depression (r = −0.480, P < 0.001), anxiety (r = −0.472, *p* < 0.001), and stress (r = −0.398, *p* < 0.001) among students during the current pandemic.

**Table 9:** Correlation analysis between the COVID-19-related stressors and the anxiety of students

Related stressors	Depression level		Anxiety level		Stress level	
	r	*p*	r	*p*	r	*p*
Concern about economic impacts	0.236	< 0.001	0.153	< 0.001	0.159	< 0.001
Concern about educational influences	0.310	< 0.001	0.150	< 0.001	0.160	< 0.001
Impact on our daily-life	0.160	< 0.001	0.163	< 0.001	0.142	< 0.001
Social support	−0.480	< 0.001	−0.472	< 0.001	−0.398	< 0.001

r Correlation coefficient

## Discussion

During the COVID-19 period, the current study aims to identify the levels of depression, stress, and anxiety among school students. Regarding gender differences in stress, both females and males seemed to suffer equally. Females had severe anxiety and depression, while males had moderate levels of anxiety and depression. This may be because females have more reactivity in fear-related neural networks and have greater differential conditioned skin conductance responses to stimulations than males [36]. Female students often experience more stress and depression than male students as a result of the fear of losing their educational accomplishments as a result of school closures during the COVID-19 pandemic, as well as the fear of COVID-19 having a negative effect on the wellbeing of their families or relatives [37]. Reasonable and moderate levels of stress, depression, and anxiety may also inspire people to participate in healthier behaviors in reaction to COVID-19, such as increasing risk tolerance and adopting preventive procedures during the pandemic [38–40]. For instance, the differences observed in this analysis between males and females may be related to risk perception. Recently, it was reported that gender was an important predictor variable of risk perception levels among respondents, where being a female was a predictor of increased risk perception.

Similar results were previously documented in a study carried out by Wang et al. [12], who found that the female gender was a predictor of the negative psychological influence of the COVID-19 pandemic, with females experiencing a greater psychological impact and higher levels of depression, stress and anxiety than males. This is also in line with other research that has shown females to be more psychologically vulnerable than males in the time of COVID-19 [1, 36, 41–43]. Liu et al. [1] investigated the prevalence and predictors of post-traumatic stress symptoms in China during the COVID-19 pandemic [36]. The results showed that females reported higher levels of post-traumatic stress symptoms in the domains of negative cognition or mood, re-experiencing, and hyper-arousal. Rossi et al. [43] also showed that the female gender was correlated with a greater psychological effect of COVID-19, as well as higher levels of stress, anxiety, depression, insomnia, adjustment disorder, and perceived stress. More recently, Hou et al. [44] found that males suffer from less extreme anxiety symptoms than females. On the other hand, Rehman et al. [5] found that females and males did not differ significantly in terms of stress, depression and anxiety.

The findings of the current study found a significant difference among the two age groups on stress, anxiety, and depression. Both age groups reported moderate depression. The older students (secondary school students) reported moderate stress and severe anxiety, whereas younger students (primary school students) reported mild stress and moderate anxiety. Secondary school students experience higher stress and anxiety than primary school students. The key reason for this seems to be that secondary school students are frequently using social media platforms, so they have a greater opportunity to obtain information with respect to COVID-19, which cause fear, panic, stress, anxiety, and depression due to the spread of rumours, fabricated news, and misinformation about COVID-19. Also, secondary school students are concerned about the consequences caused by this crisis on their educational achievements due to the closing of schools during the COVID-19 pandemic [45]. Secondary school students may be more aware of the danger of infecting with COVID-19 and have a more realistic perception of the events caused by this pandemic, so their stress and anxiety were greater. The authors explain from their experience and being teachers who work in primary schools that primary school students are not aware of the current health crisis and do not have sufficient awareness of the risk of infection with COVID-19, so they had less stress and anxiety. The authors confirmed that some primary students considered themselves on a vacation and they are doing daily activities as usual such as playing on roads, going to picnics and attending the social events during the COVID-19 lockdown.

This study also highlights how school students have suffered in the time of quarantine due to the COVID-19 pandemic [30, 46], where secondary school students reported moderate stress and severe anxiety. This result could be attributed to the closure of schools, where the students shifted from traditional learning to E-learning, therefore the academic performance of students was significantly affected, particularly those students who have limited access to the internet or students who have not a mobile phone. In contrast, students were found to be depressed to a moderate level which might contribute to changes in their lifestyle and daily activities, and in their learning activities.

According to the findings of this study, students from low-income families showed higher levels of stress, depression and anxiety. This result could be attributed to the fact that low-income families have limited access to daily necessities and materials required for preventive procedures such as gloves, face masks, hand sanitizers, and high-quality foods during the COVID-19 lockdown. As a result of lockdown and loss of work, most Palestinian households lose their daily income, therefore inability to provide the necessities to their families. Anxiety, depression, and stress became significantly higher in students from low-income families during the COVID-19 period. Individuals of those families are worried about the pandemic's long-term effects and economic hardships since they are actively working forces in communities that are disproportionately influenced by lockdown such as layoffs and loss of wages [47–49].

Students from larger families had higher levels of anxiety, depression, and stress. It was reported that there is a correlation between family size and levels of stress, anxiety, and depression [50, 51]. More recently, Le et al. [52] conducted a study during the COVID-19 era, they revealed that having a larger family is linked to higher levels of depression, anxiety, and stress [52]. This finding is attributed to increased concerns about the risk of infection of their family member [12]. Furthermore, households with large families face more financial stress.

Confirming our hypothesis, COVID-19-related stressors such as concern about economic impacts was positively correlated with anxiety symptoms in students during the COVID-19 pandemic. According to the study of Bodrud-Doza [53], McKee and Stuckler [54], Pak et al. [55] and Qin et al. [56], the pandemic have a significant influence on the country's economy and individuals, in addition to the public health crisis. Many Palestinian families lost their source of income due to lockdown policy as a result of the widespread of the COVID-19 pandemic [3], therefore students may be concerned about paying their tuition fees [57–60] or inability to continuing their distance learning due to the lack of electronic resources such as internet, mobile phone, tablet [29].

In addition, one of the COVID-19-related stressors is concern about educational influences was positively correlated with anxiety symptoms in school students during the COVID-19 pandemic. This result is expected at the time of conducting this study since preventive measures have been implemented to prevent the spread of COVID-19 among students, such as schools closures, which have been prolonged to prevent a large-scale infection among students. When schools closed, the students shifted to e-learning to complete their education through various digital platforms. Students face numerous challenges with regard to employing e-learning tools in their education, including the inability to study all subjects, complete assignments, and manage their time. Students were likewise concerned about their academic performance and achievement. Besides the negative impacts of the COVID-19 pandemic on students, the Gaza Strip is still suffering from numerous problems related to all aspects of life due to the hard blockade imposed by the Israeli occupation since 2007. Students and their families strongly struggle to be in life due to hard poverty, inability to provide the required necessities, frequent cutting off electricity, destroyed infrastructure, food insecurity, water pollution, increased unemployment rate, lost jobs, and other many problems [61–63]. Most students come from very low-income families and their guardians inability to provide them with electronic resources (laptop, tablet, mobile phone, internet,.. etc.) required to continue their education through remote learning platforms. All the above-mentioned factors put students in a hard situation when they suddenly shifted from traditional classrooms to virtual classrooms.

Also, one of the COVID-19-related stressors is its impact on daily life, as it was positively correlated with anxiety symptoms in students during the COVID-19 pandemic. This result is expected during this pandemic as schools, parks, markets and recreational places have been closed and protective procedures have been strongly imposed, particularly quarantine and isolation, so students enforced to stay home for a long period of time leading to psychological distress as stress, loneliness, anxiety, fear, depression, and panic during the COVID-19 lockdown [3, 25, 64–66]. This finding was in accordance with the finding presented in the study of Sundarasen et al. [67], Elmer et al. [68], Savitsky et al. [69] and Bourion-Bédès et al. [70], they showed that students had high levels of stress, depression, and anxiety during the lockdown COVID-19 period.

Finally, social support was found to be negatively associated with school students' anxiety, depression and stress, which is in agreement with the results reported in the study of Chen et al. [71], Cao et al. [30], Özmete and Pak [72], Grey et al. [73]‏, and Ren et al. [74]‏. They found out that social support can reduce the level of anxiety and stress of students during the COVID-9 pandemic. Social support not only decreased the level of psychological stress during the COVID-19 pandemic but also changes students’ perceptions of social support and help-seeking tactics. This finding implies that during public health emergencies, effective and powerful social assistance is required [75, 76].

## Conclusion

The levels of anxiety, stress and depression in school students were examined in this study. Throughout the early stage COVID-19 pandemic in the Gaza Strip, the results showed that the majority of the students experienced moderate to severe levels of anxiety (88.4%) and depression (72.1%), whereas less than half of them (35.7%) experienced moderate to severe stress. Because of their higher psychological distress, females, secondary school students, and those from large and low-income families need immediate care. The level of depression, anxiety, and stress symptoms of school students during the pandemic was positively correlated with COVID-19 related stressors such as economic stressors, impacts on daily living, and educational consequences, however, social support was negatively associated with the level of depression, anxiety, and stress. When faced with public health problems, school students' mental health suffers significantly, and they require the attention, assistance, and support of society, families, and schools. It is advised that the government and schools work together to solve this problem so that school students can receive high-quality, timely crisis-oriented psychological care. Our results can be used to develop a psychological intervention for students, as well as to deploy public mental health measures alongside pandemic response efforts in the early phases of an outbreak.

### Practical implications of the study

This study showed that students suffer from psychological distress during the closing school period as a result of the outbreak of COVID-19. This is an alarm for guardians, policy-makers, teachers, supervisors and principals. The psychological impacts of COVID-19 on students mental health need to be firmly placed on the agenda and priorities of responsible authorities. Besides the great attention of designing learning material and following their learning during the closing of schools, comprehensive entertainment relief programs should be designed and psychological support sessions should be prepared during back to schools. Education officials must appeal to donor countries and provide material and moral support for the implementation of these programs as our country face many financial problems. These planned programs are crucial to support the quick recovery of students after the crises, consequently contributing to enhancing students well-being.

### Limitations

This study was conducted during the emergency closing of schools due to the outbreak of the COVID-19 pandemic. In spite that this study has made an important contribution and can be used by the policymakers to address the negative psychological impacts during the COVID-19 period, it has some limitations. The study's limitations include the fact that the quarantine and lockdown periods made it difficult to directly interact with students and collect data in a systematic manner. The second limitation is related to using online Google forms for data collection that prevent students who do not have mobile phones or have limited access to internet services in the Gaza Strip. The third limitation is related to the sample size, as it is small due to the fact that that the authors were not able to reach a larger number of students since this study was carried out during the summer vacation and the closure period. In addition, the authors experienced difficulty in reaching students and inviting them to participate due to the lack of electronic resources necessary for participation (such as electricity, internet, laptop, mobile phone,.. etc.). The fourth limitation is related to the statistical nature of the study, by itself, does not provide evidence of causality between the relationships found. However, it is possible to specify that such causal relationships are plausible as they are consistent with psychological theory on these issues. We consider this study as an exploratory and descriptive study to carry out a large-scale study in the future and involve a large number of students.

## Data Availability

The data that support the results of the present study are available on request from the corresponding author. The data are not publicly available in order to respect the privacy of research participants.

## Abbreviations

MOEHE: Ministry of Education and Higher Education
MOH: Ministry of Health
